# The socioeconomic conditions of recyclers: Census data in Cali, Colombia

**DOI:** 10.1016/j.dib.2019.01.043

**Published:** 2019-01-21

**Authors:** Lina Martínez, Blanca Zuluaga, Daniela Estrada

**Affiliations:** a Universidad Icesi – POLIS, Cali, Colombia; b Universidad Icesi, Economics Department, Cali, Colombia; c Universidad Icesi, CIENFI, Cali, Colombia

**Keywords:** Recyclers, Poverty alleviation, Census data, Cali, Colombia

## Abstract

This data article describes two data sets about the socioeconomic conditions of recyclers in Cali, the third largest city in Colombia, South America. Data collected were aimed at understanding the social and economic conditions of this population in order to provide information for crafting policy alternatives for poverty alleviation. Information was collected in two waves in 2013 and in 2015. The first data collection (2013) was focused on a subgroup of recyclers and the second data collection (2015) was a census. In total, more than 3000 direct surveys to recyclers were conducted at individual and family level. In both data collection rounds, information about socioeconomic factors, health, working conditions, access to welfare programs and family composition was gathered. Both data rounds were financed by the local government as an input to design policy interventions to improve the recyclers’ quality of life in the city. The data of this manuscript is associated with the publication (Estrada et al., 2017).

**Specifications table**

**Table:** 

Subject area	Poverty reduction
More specific subject area	*Public policy formulation*
Type of data	Text, dummy, and metric variables
How data were acquired	Census – In person surveys
Data format	Raw
Experimental factors	Data described is a census. There was not random assignment component in data in this manuscript
Experimental features	There was not an experimental component in the data set
Data source location	Cali–Colombia
Data accessibility	http://repository.icesi.edu.co/biblioteca_digital/handle/10906/82189
Related research article	Estrada, Daniela., Martínez, Lina., Zuluaga, Blanca. (2017). Detrás de la carreta ¿cómo viven los recuperadores ambientales de Cali?. Universidad Icesi, Cali, Colombia.

**Value of the data**

• Data collected in both studies allow a deep understanding of the living conditions of recyclers and the levels of poverty they live in. Both studies were crafted in order to provide specific information for policy design in terms of risk factors identification and sub-population targeting to prioritize attention to the most needed. In the Colombian context, recyclers are the neediest and poorest population.
• In these data sets is possible to link individual recyclers with their families. Information about nuclear and extended family members was collected on issues related to health, education, employment, disability and illicit drugs consumption.
• At the family level, detailed information about children between zero and five years old was gathered. This information was aimed at providing information about early childhood practices, care and child placement in early programs. Likewise, for family members between 12 and 17 years old, information about early pregnancy was collected.
• One of the possibilities of these data is to build an occupation profile of recyclers. Information about skills, educational attainment, and past employment was collected. This information was aimed at providing information to the local government about alternative occupations for recyclers.
• Recyclers reported the location where they collected recycling material. This information allows us to map their activity and trace the spatial dimension of this occupation.

## 1 Data

Data presented were collected by direct surveys (face-to-face) to recyclers in Cali–Colombia in two different years. The first data collection was conducted during 2013. The second, was conducted in 2015. For both rounds, a structured survey was designed and collected by trained pollsters. The questionnaire was designed by the research team and approved by local authorities. In both studies, recyclers answered questions regarding their socioeconomic background, housing characteristics, working conditions, family composition, income, employment profile and skills, education, life satisfaction, and access to government welfare. The overall characteristics of recyclers are presented in Table 1.

**Table 1:** Overall Characteristics of the Sample, Cali – 2013 and 2015.

Characteristic	Year	
	2013 (*n* = 1603)	2015 (*n* = 3109)
**Demographics and income**		
Men (%)	60.9	51.9
Women (%)	39.1	48.1
Illiteracy rate (%)	21.4	17.3
Average schooling years	4.4	4.8
Less than 1 minimum wages of monthly income^a^ (%)	80	87
**Marital status**		
Married (%)	9.1	8.5
Living common law (%)	44.8	35.5
Single (%)	35.4	42.8
Divorce (%)	6.6	8.8
Widower (%)	4.1	3.9
**Race/Ethnicity**		
Indigenous (%)	10.9	8.4
Black (%)	30.1	40.8
Mestizo (%)	39.2	32.5
White (%)	11.3	13.3
None (%)	8.0	5.0
**Household conditions**		
Own household (%)	25.6	22.4
Toilet not connected to sewer (%)	8.6	9.3
Prevailing material of the floors of the dwelling: sand and soil (%)	NA	8.2
Overcrowding (%)^c^		28.9
**Family composition**		
Size (average number of members)	3.7	3.9
Dependency ratio	54.8	50.5
Health insurance (%)	80.0	72.2
Illiteracy rate (%)	13.0	12.9
Less than 1 minimum wages of monthly income (%)^b^	78.6	78.0
Parent (%)	85.6	85.3
Mean number of children (%)	3.0	3.0
**Health**		
Health insurance (%)	77.4	69.5
Felt physically ill over the last 30 days (%)	NA	47.9
Felt mentally ill over the last 30 days (%)	NA	30
Mean days per month felt physically ill	NA	12.6
Mean days per month felt mentally ill	NA	13.6
**Job conditions**		
More than 5 years being a waste picker (%)	80.9	77.0
Picks material in public road (%)	NA	87.7
Use any personal protection when picking trash (%)	NA	28.6
Transportation mean used to recycle trash: bicycle/ tricycle (%)	67.9	57.4

a Aged over 25 years old.

b Aged over 12 years old.

c Three or more persons per room.

The questionnaire used in 2013 included 59 questions, from which 160 variables were systematized. On average, it took about 15–20 min to complete this survey. In 2015 greater resources were allocated in order to conduct a census of this population. Questionnaire structure was similar to the one used in 2013, although some additional information on work habits and occupational profile were included. Thus, the questionnaire had 79 questions from which 210 variables were systematized and took about 35–40 min to complete. Raw data for the 2015 census is annexed as Supplemental material. This data set is anonymized and sensible information is not provided. Questionnaires used in 2013 and 2015 are presented as Supplemental material of this publication.

Analysis of these data are published in a publicly available book called “Detrás de la carreta” [1], that can be accessed through this link.

## 2 Experimental design, materials and methods

One of the most challenging factors of this project was implementing a strategy for data collection. Recycler move around the city based on the time and routs of garbage collection settled by waste collection companies that operate in the city. Recycler has to arrive before the waste collection trucks gather the recycling materials. Given the informality of this occupation, it is not possible to know ex-ante which areas of the city they cover and what days they work. Our targeted population work in hours (late at night or very early before sunrise) that do not allow safe and feasible conditions for data collection. Besides safe conditions and work schedule, conducting the surveys in the places where they conduct their job could lead to duplication (doing the survey more than once for the same person) or missing observations (not doing the survey).

Surveys at households were also ruled out for several reasons. First, the government did not have accurate records of household location. Second, garbage collection is an occupation with a greater instability (people enter and leave of this occupation often), meaning that resources could be spent in locating a person who is no longer a trash picker. Third, this population lives in the most impoverished and unsafe areas of the city, generating a great risk for pollsters.

Taking into account all these conditions, the research team in conjunction with the local government, agreed on having central points for data collection.

In 2013, three places where selected based on closeness to the areas where the targeted population live. Recyclers live in three areas of the city: the west (Agua Blanca district), downtown (El Calvario and Sucre), and hillside (districts 1, 18 and 20). The research team used three places in the city (south, west, north) to apply the survey to recyclers. Before field work, advertisements such as posters, flyers and media announcements (radio stations and free local newspapers) were used to make public the purpose of the data collection and to let know the benefits for recyclers to participating. Other strategies for spreading out the word was sending information to recyclers’ associations and informing key leaders.

Field work lasted one week. During that time, recyclers could choose any of the places at any time from 8 am to 6 pm to participate. Additionally, other places were adapted in three of the neighborhoods where the majority of recyclers are located (downtown and hillside). Moreover, pollsters went to several ‘bodegas’ (places where recyclers sell the collected material) to obtain information from those who did not go to the data collection places. Using these strategies, 1.603 observations were collected.

In 2015, local authorities had to take measures for responding to a Supreme Court ruling (Auto 118/14 (2014)). Under this ruling, the local government had to update the information of the entire population of recyclers, including those living in urban and rural areas. The same research team was hired. In order to cover the entire city and provide recyclers with more alternatives to be counted, 22 places for permanent data collection sites were facilitated. The city has 22 districts and each district has a center for integrated local attention (C.A.L.I. for its initial letters in Spanish). Those centers are created aiming at providing information to habitants about government programs and facilitating processes related with local authorities. As the data collection conducted in 2013, a large call was used to inform about the census providing details about places for data collection, schedules, and requirements (recyclers had to present their national ID for data collection). In addition, data were collected in all the 15 rural areas of the city.

Over 80 pollsters were trained receiving intensive training about data collection and language to be used during the survey. Likewise, there were field supervisors in each of the collection data places. Besides field supervisors, other strategies were used to ensure data quality. During data collection, when an inconsistency was detected, pollsters informed supervisors to re-call recyclers to correct the information missed or inconsistent. As an additional strategy, a verification process was implemented. 10% of the population surveyed was randomly selected for household visits. During the visit, it was revised the accuracy of the information provided. All these strategies were orientated to guarantee the quality of the databases. In this census, 3.109 recyclers were surveyed. Information was used for policy making formulation.
