# Supplementary material for: The socioeconomic conditions of recyclers: Census data in Cali, Colombia
Source: Data Brief. 2019 Jan 21;23:103695. doi: 10.1016/j.dib.2019.01.043 (PMC6369313; doi:10.1016/j.dib.2019.01.043)
Supplement: Supplementary file 4 — Supplementary material [file mmc4.pdf]

E0001	UNIVERSIDAD ICESI · POLIS Observatorio de Políticas Públicas ICESI	**2009 CENSUS VERIFICATION 2013**	ALCALDÍA DE SANTIAGO DE CALI

Date ______ day ______ month ______ year ______

Interviewer's name:

2009 Census verification

1.ID number:
2. First name:
3. Last name:
4. City

5. Were you polled in 2009? **Yes** 1 [ ] **Pass to question 6** **No** 2 [ ] **Pass to question 7**

6.Unique Recycler ID (R.U.R)? ____________ **Pass to question 8**

7. Why weren't you surveyed?
1 [ ] I was not a recycler
2 [ ] I was not in the city
3 [ ] I was a recycler but did't know about the census
4 [ ] I was sick or busy
5 [ ] I didn't want to participate
6 [ ] Other Why? ____________

8.Address
9. Telephone or cellphone
10.District:
11. District's name:
12. Socioeconomic strata:

13.Zone:
Rural [ ] 1 Urban [ ] **2**

14.Do you belong to any recycler's association? **Yes** 1 [ ] **No** 2 [ ]
Association's name ____________

II. SOCIODEMOGRAPHIC CHARACTERISTICS OF THE RECYCLER

15. Gender **Male** 1 [ ] **Female** 2 [ ] **Transgender** 3 [ ]

16. Age : ____________

17.How many children do you have? [ ] **18.How many of your children live with you?** [ ]

19. Place of birth (municipality): ____________

20. How many years have you been living in Cali? **Number of years** [ ]

21. Have you lived in another city? **Yes** 1 [ ] **Pass to question 22** **No** 2 [ ] **Pass to question 23** **Which city?** ______

22. What was the main reason to change municipality of residence?
Forced displacement [ ] 1 Looking for a job [ ] 2 Searching for better opportunities [ ] 3
Doesn´t know / Doesn´t answer [ ] 4 Other [ ] 5 Which reason? ____________

III. FAMILY, HOUSEHOLD AND EXPENSES

23. Including housing, food, services and others, the monthly expenses of your home are approximately: ____________ **daily** [ ] 1 **monthly** [ ] 2

24. Type of household
[ ] 1. House [ ] 2. Room [ ] 3. Shack
[ ] 4. Apartment [ ] 5. Tenement house [ ] Other

25. What is the prevailing material of the dwelling's floor?
[ ] 1. Floor (include Carpet, marble, polished or lacquered wood , Tile, vinyl, tablet, brick , Cement, gravel ,Coarse wood, board, plank, other vegetable source
[ ] 2. Soil, sand

26. What is the prevailing material of the dwelling´s walls?
[ ] 1. Block, brick, stone, polished wood, Tapia pisada, adobe, bahareque, Coase wood, board, Prefabricated material
[ ] 2. Guadua, cania, mat, other vegetable sources,Zinc, cloth, cardboard, cans, scraps, plastics,No walls

27. How many rooms does your household have? (do not include bathrooms and kitchen)
Number ____________

28.What type of toilet does the household have?
1 Toilet connected to sewer
2 Toilet connected to septic tank
3 Toilet without connection
4 Latrine
5 It does not have

**29. Your dwelling have access to:**	**YES(1)**	**NO(2)**
1. Aqueduct		
2. Energy		
3. Gas		
4. Sewerage		

30. Your household is
1.Your own [ ]
2.Rented [ ]
3. From a family member [ ]
4. Other [ ] Specify: ____________

31. During the last twelve months, did a member of this household receive subsidies, either in cash or in kind, from government entities for:

	**YES(1)**	**NO(2)**	**How many members receive it?**
1. Familias en acción			
2.Elderly subsidy			
3. Other			

Which subsidy?________________________

E0001	UNIVERSIDAD ICESI · POLIS Observatorio de Políticas Públicas ICESI	2009 CENSUS VERIFICATION 2013	ALCALDÍA DE SANTIAGO DE CALI

Date ______ day ______ month ______ year ______

IV. WORKING CONDITIONS AND OCCUPATIONAL PROFILE OF THE RECYCLER

33. Are you currently working as a recycler?

Yes 1 [ ] No 2 [ ] **Pass to question 52**

34. How long have you been a recycler?

1. Less than 5 years [ ]
2. From 6 to 10 years [ ]
3. From 11 to 20 years [ ]
4. From 21 to 30 years [ ]
5. From 31 to 40 years [ ]
6. More than 40 years [ ]

35. Would you like to change your job?

Yes 1 [ ] No 2 [ ] **Why?** ______

36. What type of material do you recover?

______

37. Where do you recover?
Districts: ______

38. Mention 5 neighborhoods where you recover frequently

______

39. Where do you sell the the recovered material?

Name of warehouse ______ Address ______
Name of warehouse ______ Address ______

40. On average, how much do you earn for the material you sell? ______

41. On average, how many kilos of material do you recover per day? ______

42. On average, how many days per week do you work? ______

43. Which days of the week do you work?

1 [ ] Monday
2 [ ] Tuesday
3 [ ] Wednesday
4 [ ] Thursday
5 [ ] Friday
6 [ ] Saturday
7 [ ] Sunday

44. On average, how many hours a day do you work? ______

45. Indicate your working time in a normal day ______ ______

46. Without subtracting daily payments,what is your gross daily income? ______

47.What means of transportation do you use to pick material?

1. Own car [ ]
2. rented cart [ ]
3. Sack, bag [ ]
4. Other [ ] Which? ______

48. Aside from working as a recycler, what other activity do you do?

1.None, dedicated exclusively to work as a recycler [ ]
2.Another job [ ] Which job? ______
3. Another non-work activity [ ] Which? ______
4. . Looking for a job [ ]

49. Working activity performed before being a recycler: ______ **Not apply** [ ]

50.Do you drive a vehicle?

1. Car [ ]
2. Motorcycle [ ]
3. Bicycle [ ]
4. Doesn´t drive any vehicle [ ]

51.Do you have a driving license?

1. Yes [ ] Category ______
2. No [ ]

52. What is your actual occupation? (Only for those who answered No to question 33) ______

53. Have you received training or informal education?

Yes 1 [ ] No 2 [ ] **Pass to question 59**

54. In what area did you receive training or informal education? (Read options)

1 [ ] Debris management
2 [ ] Recycling
3 [ ] Solid Waste Management
4 [ ] Building
5 [ ] Automotive mechanic
6 [ ] Joinery or carpentry
7 [ ] Electricity
8 [ ] Metalworking
9 [ ] Basic Environmental Legislation
10 [ ] Maintenance of green areas
11 [ ] Clothing or dressmaking
12 [ ] Culinary, bakery or biscuit
13 [ ] Shoe shop
14 [ ] farming
15 [ ] Other ______

55. Do you consider that the training you received has been applied?

Yes 1 [ ] No 2 [ ]

56. Did the training you received allow you to get another job?

Yes 1 [ ] No 2 [ ]

57. Would you like to receive more training workshops?

Yes 1 [ ] **Pass to question 58**
No 2 [ ] **Pass question to 59**

58. In which area would you like to receive training? . Select a maximun of 3 options. (Read options)

1 [ ] Debris management
2 [ ] Recycling
3 [ ] Solid Waste Management
4 [ ] Building
5 [ ] Automotive mechanic
6 [ ] Joinery or carpentry
7 [ ] Electricity
8 [ ] Metalworking
9 [ ] Basic Environmental Legislation
10 [ ] Maintenance of green areas
11 [ ] Clothing or dressmaking
12 [ ] Culinary, bakery or biscuit
13 [ ] Shoe shop
14 [ ] farming
15 [ ] Other

59. Do you feel you are capable of doing the following activities?

	Yes	No
1 Public speaking		
2 Managing personal		
3 Managing money		
4 Training or teaching other people		
5 Managing a business		
6 Plannig activities that involve other individuals		

END

Observations

______

Thank and finish

SIGNATURES The signatures and footprints below do not imply any contract between the signatories, it only serves to corroborate the identity of participants

Handprint - right index finger ______ Recycler ______ Interviewer/Pollster ______ Superviser
