# Supplementary material for: The socioeconomic conditions of recyclers: Census data in Cali, Colombia
Source: Data Brief. 2019 Jan 21;23:103695. doi: 10.1016/j.dib.2019.01.043 (PMC6369313; doi:10.1016/j.dib.2019.01.043)
Supplement: Supplementary file 5 — Supplementary material [file mmc5.pdf]

E0001

UNIVERSIDAD ICESI | POLIS Observatorio de Políticas Públicas ICESI

RECYCLERS 'CENSUS VERIFICATION AND UPDATE OF 2009 CENSUS, BY AUTO 118 OF 2014

ALCALDÍA DE SANTIAGO DE CALI

Date: day ____ month ____ year ____ Zone: ____ Hour: ____

Interviewer's name:

2009 Census verification

1.ID number:

2. First name:

3. Last name:

4. Place of birth (municipality):

5. Were you polled in 2009? Yes 1 ☐ Pass to question 6 No 2 ☐ Pass to question 7

6. Unique Recycler ID (R.U.R)? ____________ Pass to question 8

7. Why weren't you surveyed?

1 ☐ I was not a recycler
2 ☐ I was not in the city
3 ☐ I was a recycler but did't know about the census
4 ☐ I was sick or busy
5 ☐ I didn't want to participate
6 ☐ Other Which?

8. Do you belong to any recycler's association?

Yes 1 ☐
No 2 ☐ Pass to question 12

9. Association's name ____________

10. How long have you been a member of the association? ____________

11. Type of participation you have in the association

1 ☐ Active member
2 ☐ Inactive member

I. RECYCLER SOCIODEMOGRAPHIC CHARACTERISTICS

12. Home address ____________

13. District: ____________

14. Socioeconomic strata: ____________

15. Telephone number ____________ Belongs to
1 ☐ Recycler
2 ☐ Other person

16. Cell phone number ____________ Belongs to
1 ☐ Recycler
2 ☐ Other person

17. How many years have you been living in Cali?
Num. of years ☐ Num. of months ☐

18. Date of birth Day ____ Month ____ Year ____

19. Gender
1 ☐ Male
2 ☐ Female
3 ☐ Transgender

20. Do you have children?
1 ☐ Yes 20.1 How many? ____
0 ☐ No (Pass to question 23)

21. How many of your children live with you? ____________

22. How old were you at your first childbearing? ____________

23. Including you, how many people live in your household? ____________

24. Are you the head household?
1 ☐ Yes
0 ☐ No

25. How many people contribute with economic resources to your household? (Including yourself) ____________

II. HOUSING CONDITIONS AND HOME SERVICES

26. Type of dwelling
1 ☐ House
2 ☐ Room
3 ☐ Shack
4 ☐ Apartment
5 ☐ Tenement house
6 ☐ Other
26.1 Which? ____________

27. Your household is
1 ☐ Your own it but still paying the loan
2 ☐ Your own
3 ☐ Rented
4 ☐ Other
27.1 Specify ____________
Pass to question 29

	28.1.Do you have a housing subsidy ?	28.2 Did you receive the subsidy for being a recycler? Yes (1)	No (0)
(1) Yes			
(0) No		Not apply	

29. Prevailing material of the dwelling's floor?
1 ☐ Carpet, marble, polished or lacquered wood
2 ☐ Tile, vinyl, tablet, brick
3 ☐ Cement, gravel
4 ☐ Coarse wood, board, plank, other vegetable source
5 ☐ Soil, sand

30. Prevailing material of the dwelling's walls?
1 ☐ Block, brick, stone, polished wood
2 ☐ Tapia pisada, adobe, bahareque
3 ☐ Coase wood, board
4 ☐ Prefabricated material
5 ☐ Guadua, cania, mat, other vegetable sources
6 ☐ Zinc, cloth, cardboard, cans, scraps, plastics
7 ☐ No walls

31. How many rooms does your household have? (do not include bathrooms and kitchen)
Number ____________

32. How many rooms are used to sleep?
Number ____________

33.What type of toilet does the household have?
1 ☐ Toilet connected to sewer
2 ☐ Toilet connected to septic tank
3 ☐ Toilet without connection
4 ☐ Latrine
5 ☐ It does not have

34. Your dwelling have access to:

	Yes(1)	No(0)
1. Aqueduct		
2. Energy		
3. Gas		
4. Sewerage		
5. Garbage collection		

35. The water to prepare food is obtained mainly from:
1 ☐ Public aqueduct
2 ☐ Communal or veredal aqueduct
3 ☐ Well with pump
4 ☐ Well without pump, jagüey
5 ☐ Water rain
6 ☐ River, stream or mountain spring
7 ☐ Public stack
8 ☐ Water tank car
9 ☐ Water carrier
10 ☐ Bottled or bagged water

III. FAMILY

36. Data of the cohabiting family (include exclusively the members with whom the person surveyed lives)

The table INCLUDES the person being surveyed: fill in only the boxes marked with an "*" asterisk.

III FAMILY

Code	Field	1	2	3	4	5	6	7	8
36q	* Drugs Consumption (1= Yes 0= No)								
36p	* Disability (Type)								
36o	*Average monthly income in current job								
36n	*Contribute to a pension fund (1=Yes 0=No)								
36m	* What type of health insurance do you have?								
36l	*(Ethnicity) According to your culture, physical features you recognize yourself as:								
36k	*Last year approved in that educational level								
36j	Educational level								
36i	* Are you currently studying? (1= Yes, 0= No)								
36h	* Do you know how to read and write? (1= Yes, 0= No)								
36g	* Marital Status								
36 f	Unemployed time (only if 36e = 2)								
36e	Actual occupation								
36d	Kinship	Do not fill these boxes for the recycler							
36c	Gender (1=Man, 2=Woman, 3=Other)								
36b	Age								
36a	Last Name		1 / 2	1 / 2	1 / 2	1 / 2	1 / 2	1 / 2	1 / 2
36a	First Name		1 / 2	1 / 2	1 / 2	1 / 2	1 / 2	1 / 2	1 / 2
	Member order	1	2	3	4	5	6	7	8

36d Family kinship

1. Spouse or partner
2. Son/Daughter
3. Step-son/ step daughter
4. Grandchild
5. Father or mother
6. Brother or sister
7. Son-in-law ir daughter-in-law
8. Grandfather/grandmother
9. Father-in-law/mothe
10. Uncle and aunt
11. Nephew
12. Cousin
13. Brother-in-law/sister-in-lav
14. Other

36e. Actual occupation

0. No activity
1. Worker
2. Unemployed
3. Student
4. Rentier
5 Retired
6. Housekeeper

36g Marital Status

1 Common law marriage
2 Married
3 Widowed
4 Divorced
5 Single
6 It doesn't apply

36j Highest educational level

0 None
1 Preschool
2 Complete primary
3 Incomplete primary
4 Complete secondary
5 Incomplete secondary
6 Technical or technology
7 Undergraduate degree

36k. Last year aproved

Elementary education : from 1 to 5 years
Secondary Education: from 6 to 11 years
Technical or techonology: from 1 to 3 yea
Higher education: from 1 to 5 years

36l. Ethnicity

1 Indigenous
2 Black
3 Mestizo
4. White
5. Gympsy
6. None

36m. Health insurance

1.Employee contributor
2. Independent contributor
3. He/she is affiliated to an EPS of the Subsidized Regime
4. He/she is a beneficiary of an employee or pensioner affiliated to an EPS
5. Belongs to an indigenous reservation
6. Although you have a SISBEN card, you do not have EPS of the Subsidized Regime
0. None

36o. Average monthly income in current job

1. Less than 1SMMLV
2. Between 1 and 2 SMMLV
3. More than 2 SMMLV
4 No income
5. Not apply

36p. Type of disability

1. Sensory
2. Mobility Impairments.
3. Cognitive
4. Mental
5. Multiple
6. No disability

E0001	ICESI / POLIS	RECYCLERS 'CENSUS VERIFICATION AND UPDATE OF 2009 CENSUS, BY AUTO 118 OF 2014	ALCALDÍA DE SANTIAGO DE CALI

IV. CARE OF CHILDREN 0-5 YEARS OLD

37. Are there household members under 5 years old (<= 5)? Interviewer answer this question by looking at table on page 2, do not ask.

1 [ ] Yes 37.1 How many? ______ 0 [ ] No (Pass to question 39)

38. Where or with whom do children stay most of the time during the week?

Member number from page 2	Answer	8 Other / Which?

Response options:
1 Attend a community home, nursery or garden
2 With its father or mother in the house
3 With its father or mother at work
4 With the maid or nanny in the house
5 In care of a relative 18 years or older
6 In care of a relative younger than 18 years
7 Alone in the house
8 Other

V. CHILD LABOR AND ADOLESCENT PREGNANCY

FOR HOUSEHOLD MEMBERS BETWEEN 12 AND 17 YEARS

39. Are there household members between 12 and 17 years old? Interviewer answer this question by looking at table on page 2, do not ask.

1 [ ] Yes 39.1 How many? ______ 0 [ ] No (Pass to question 41)

40. About members from 12 to 17 years old

Member number from page 2	40.2 Already a parent		40.3 How many children?	40.4 Is she currently pregnant? If ithe member is a man," Is his partner currently pregnant? "		40.5 Activity that he/she performs most of the time	40.6 Type of job	40.6.1 Other / which?
	Yes(1)	No(0)		Yes(1)	No (0)			

40.5 answer options:
1 Work
2 Study and work
3 looking for a job
4 Study
5 Household chores
6 None of the above

(4–6: Pase a la pregunt a 41)

40.6 answer options:
1 Recycling
2 Domestic service
3 Sales
4 Street sales
5 Construction
6 Other

VI. INCOME AND EXPENSES

41. Without subtracting daily payments, how much is your daily income? ______ [ ] Daily (1) [ ] Monthly (0)

42.Including housing, food, services and others, the monthly expenses of your home are approximately:

Value	Daily (1)	Monthly (0)

43. During the last twelve months, a member of this household received subsidies, either in cash or in kind, from government entities for:

	Yes(1)	No(0)	Num. Of member
1. Cash transfer			
2.Elderly subsidy			
3. Other			

43.3 Which ______

44. What have you done during your life to ensure a good life when you get older?

(Multiple answer)

1 [ ] Expects that children will assume the responsibility
2 [ ] Save money for the future
3 [ ] Try to organize a business / asset that guarantees an income
4 [ ] Listed in a pension fund
5 [ ] Hopes that someday he/she will get the money to save for old age.
6 [ ] Nothing
7 [ ] Other 44.1 What ______

VII. LIFE SATISFACTION

45. On a scale of one to ten, rate how satisfied you are with your life. (If you answer 10 skip to question 47)

Number [ ]

46. What's necessary to be completly satisfied with your life? (Spontaneous responde - multiple, maximum 3 options, enumerate the order of response)

( ) 1 [ ] Good health
( ) 2 [ ] Money
( ) 3 [ ] Own household
( ) 4 [ ] A stable relationsh
( ) 5 [ ] A job
( ) 6 [ ] Family
( ) 7 [ ] Better family relationships
( ) 8 [ ] Study
( ) 9 [ ] Professional or personal success
( ) 10 [ ] A better physical aspect
( ) 11 [ ] Free time
( ) 12 [ ] A car/own means of transporte
( ) 13 [ ] Security
( ) 14 [ ] Family health
( ) 15 [ ] Other, what? ______

VIII. SALUD

47. Your health status is

1 [ ] Excellent
2 [ ] Very good
3 [ ] Good
4 [ ] Regular
5 [ ] Bad
6 [ ] Do not know / Do not answer

48. Now think about your physical health, which includes physical illnesses and accidents, how many of the past thirty days did you not enjoy good physical health?

Number of days ______

99 [ ] Do not know / Do not answer

49. Now think about your mental health, which includes stress, depression and emotional problems: For how many of the past thirty days did you not enjoy good mental health?

Number of days ______

99 [ ] Do not know / Do not answer

50.Now I'm going to ask you some questions about your physiognomy.

1. Height (m) ______ 2. Weight (kg) ______

3. Do you feel comfortable with your weight? 1 [ ] Yes 0 [ ] No

51.During the last year, do you have to be hospitalized for some reason?

1 [ ] Yes 51.1 Num. Of times ______
0 [ ] No

52. Are you affiliated to an occupational risk manager insurer (ARL)?

1 [ ] Yes
2 [ ] No
3 [ ] Do not know

E0001	UNIVERSIDAD ICESI · POLIS Observatorio de Políticas Públicas ICESI	RECYCLERS 'CENSUS VERIFICATION AND UPDATE OF 2009 CENSUS, BY AUTO 118 OF 2014	ALCALDÍA DE SANTIAGO DE CALI

IX. WORKING CONDITIONS AND OCCUPATIONAL PROFILE OF THE RECYCLER

53. Are you currently working as a recycler? Yes 1 ☐ **Pass to question 54** No 0 ☐ **Pass to question 75**

54. How long have you been a recycler?
1 ☐ Less than 5 years (**pass to question 56**)
2 ☐ From 6 to 10 years
3 ☐ From 11 to 20 years
4 ☐ From 21 to 30 years
5 ☐ From 31 to 40 years
6 ☐ More than 40 years

55. Where did you recycle before 2009? 1 ☐ Navarro 2 ☐ Street 3 ☐ Other **55.1** Where? ____________

56. Would you like to change your job? 1 ☐ Yes 0 ☐ No

57. PASS TO PAGE 5

62.Specify the route you take on each working day

		62.1 Monday	62.2 Tuesday	62.3 Wednesday	62.4 Thursday	62.5 Friday	62.6 Saturday	62.7 Sunday
Start Neighborhood								
In between neighborhoods	Neighborhood 1							
	Neighborhood 2							
	Neighborhood 3							
	Neighborhood 4							
	Neighborhood 5							
End neighborhood								

63. Where do you gather the material?
1 ☐ Public road
2 ☐ Residential
3 ☐ Mall
4 ☐ Office
5 ☐ Industry
6 ☐ Other 63.1 Where? ____________

64. Where do you sell the recycled material?
1 ☐ Warehouse
2 ☐ Business
3 ☐ Truck
4 ☐ Another recycler
5 ☐ Recycler association
6 ☐ Other 64.1Which? ____________

65. Do you use your home as a temporary place for storing the recycled material? 1 ☐ Yes 0 ☐ No

66.On average, how many hours a day do you work? ____________

67. Between which hours of the day do you work? ____________ ____________

68. On a working day, how many hours (on average) do you take on the following activities?

	Hours		Hours		Hours
1 Gathering		3 Transportation		5 Selling	
2 Collection		4 Selection		6 Material transformation	

69. What elements of personal protection do you use in your work? (Mark with an X)

	Gloves	Snap	Boots	Caps	Glasses	Overalls	Other	None
Yes (1)								

70. Do you work in company?
1 ☐ Yes
0 ☐ No

70.1 With whom?
1 ☐ Children
2 ☐ Couple
3 ☐ Other family members
4 ☐ Friends

71. Number of people that depend economically on the activity that you exercise as a recycler ____________

72. Earning for material recovery in the previous week ____________

73.What means of transportation do you use to pick material and what type of property do you have over them?

	Yes(1)	No (0)	73.2 Type of property
**Motor vehicle**			
**Animal traction**			
**Carreta**			
**Tricycle/bicycle**			
**Sack, bag**			
**Other Which?**___________			

73.2 answer options
1 Your own
2 Rented
3 Communal property
4 Borrowed
5 Other

74. besides working as a recycler, what other activity do you do?
1. None, exclusively dedicated to work as a recycler ☐
2. Another job ☐ Which job? ____________
3. Another non-working activity ☐ Which activity? ____________
4. Looking for a job ☐

75. Work activity performed before being a recycler: ______________________________ **Not apply** ☐

76.Do you drive a vehicle? (Multiple answer)
1. Car ☐
3. Bicycle ☐
2. Motorcycle ☐
4. Do not drive ☐

77. Do you have a driving license?

	Yes (1)	No (0)
Motorcycle		
Car		

78. What do you do now? (Only for those who answered No to question 53) ____________

79. Have you received training in solid waste management? 1 ☐ Yes 0 ☐ No (end the questionnaire)

. Who provided you with the latest training in solid waste management? ______________________________

END

Observations

**Check the interview (check X)**	Complete		Incompleted		Rejected	

Signature

The signatures and handprints below do not imply any contract between the signatories, only serve to corroborate the identity of the people and are necessary to validate the file. In addition they indicate that you have received the certificate with the consecutive form that has been applied, which will serve as a precarnet for the subsequent processes of carriage by the municipality. If you have not received the certificate, do not sign it.

Handprint - right index finger

Recycler

Interviewer/Pollster

Superviser

IX. WORKING CONDITIONS AND OCCUPATIONAL PROFILE OF THE RECYCLER

P59 Answer options
1 Dry
2 Wet
3 Washed
4 Oiled
5 Mixed

After you finish this section **PASS TO QUESTION 62 (PAGE 4)**

Material	TOTAL PAPER	White	Archive	Directory	Magazine	Newspaper	TOTAL PAPERBOARD	KRAFT	Corrugated	Folding	TOTAL GLASS	Transparent glass	Vamber glass	Green glass	TOTAL PLASTIC	PET	PEAD	PVC	PEBD	PP	PS	TOTAL SCRAP METAL	Aluminio	Cobre	RCD	TOTAL SCRAP NO FERROUS	TOTAL WOOD	TOTAL OTHERS
		Paper						Paperboard				Glass				PLASTIC							SCRAP METAL					
**61. Address where you sell the material**																												
**60. On average, how much do you you get receive per day for the material you sell (Price ($ / day))?**																												
**59. What is the quality of the recovered material?**																												
**58.How many kilos per day do you recover on average (kg / day)?**																												
**57. What material do you recover and what benefit do you give to it?** — **Mark with an X the benefit i**: Other benefit / Which?																												
Trituration																												
Transformation																												
Torn																												
Compaction																												
Cleaning																												
Classification																												
Selection																												
He/she gather it (anwer which benefit does he/she gives it)																												
He/she doesn't gather it																												
